# A Simplified Model for Shear Behavior of Mortar Using Biomimetic Carbonate Precipitation

**DOI:** 10.3390/ma16165613

**Published:** 2023-08-13

**Authors:** Yu Diao, Jitao Bai, Changyou Sun, Jianyou Huang, Chao Yang, Qingsong Hu

**Affiliations:** 1 School of Civil Engineering, Tianjin University, Tianjin 300072, China; 2 3rd Construction Co., Ltd. of China Construction 5th Engineering Bureau, Changsha 410021, China; 3 China State Construction Engineering Corporation, Beijing 100029, China

**Keywords:** sand reinforcement, L-aspartic acid, biomimetic carbonate precipitation (BCP), direct shear test, simplified model

## Abstract

As a common molecule in biomineralization, L-aspartic acid (L-Asp) has been proven to be able to induce in vitro CaCO_3_ precipitation, but its application in sand reinforcement has never been studied. In this study, L-Asp was employed in sand reinforcement for the first time through the newly developed biomimetic carbonate precipitation (BCP) technique. Specimens with different number of BCP spray cycles were prepared, and a series of direct shear tests were conducted to investigate the impact of spray number on shear strength, critical displacement, and residual strength. Then a simplified power model for shear stress–displacement behavior was established and calibrated with the measured data. The results show that BCP can significantly improve the shear strength of sand. As the number of spray cycles increases, both the shear strength and residual strength increase, while the critical displacement decreases. Such variations can be described with two sigmoid models and a linear model, respectively. The simplified power model performs well in most cases, especially at higher spray numbers. This study is expected to provide a practical model for the shear behavior of BCP-treated mortar.

## 1. Introduction

Sand reinforcement is a common practice in geotechnical engineering. Conventional techniques are usually achieved with cement-based materials [1]. However, the production of cement generates plenty of pollutants like NO*_x_*, SO_2_, and particulates [2], especially CO_2_ [3] that could significantly contribute to the greenhouse effect. Therefore, other alternatives like bio-cementation techniques are drawing more and more attention. The common mechanism of those bio-techniques is to form CaCO_3_ in sand. In this sense, other chemicals that can induce a CaCO_3_ precipitate also have the potential to get sand reinforced.

Aspartic acid (Asp) and its enantiomers, including L-aspartic acid (L-Asp) and D-aspartic acid (D-Asp), have long been found to be able to mediate CaCO_3_ crystallization. Actually, Asp is a common component of biomacromolecules responsible for biomineralization [4,5]. Studies have shown that Asp is a broad-spectrum mediator that can act on almost all phases of calcium carbonate in vitro. It can stabilize and extend the lifetime of amorphous calcium carbonate (ACC) [6,7], increase the hardness of calcite single crystals [8], and even promote or inhibit aragonite precipitation under certain conditions [9,10]. However, the most reported effect of Asp is that it can induce the formation of vaterite [11,12], even though this is usually thermodynamically disfavored. The interaction between Asp molecules and calcium has been widely investigated from the perspective of surface energy [13] and kinetics [14], but the actual mechanism is still not that clear. One generally accepted fact is that there exists a complexation effect between Asp molecules and calcium ions [15], and some functional groups like -COO^-^ could serve as potential nucleation sites for the crystallization of CaCO_3_ [16]. Recent studies show that Asp may also bind to the precipitated crystals [17] and affect the morphology, phase, and crystal structure through the additional electrostatic interactions of side-chain groups with mineral surfaces [18]. In fact, the stabilization of vaterite has been supposed to be related to the acidic residues in Asp [19]. It should be noted that the interaction between Asp and calcium is never a simple process involving only the two substances. More and more evidence has revealed that such an interaction could be affected by many factors, even the structure of water [20].

As the enantiomers of Asp, L-Asp and D-Asp have exhibited similar effects on the precipitation of calcium: both of them can induce the formation of thermodynamically unstable vaterite [21,22]. Meanwhile, the morphology as well as the stability of the precipitated vaterite are heavily influenced by the concentration of amino acids [23,24]. In addition, L-Asp has been found to play an important role in the formation of prenucleation clusters [25] and dissolution of calcite [26,27]. Like Asp molecules, the enantiomers, or exactly their carboxyl sites, can form coordinated bonds with calcium ions [28]. Therefore, they can also serve as soft templates for the synthesis of a CaCO_3_ precipitate [29]. There is an assumption that the enantiomers may also affect carbonate precipitation through selective adsorption on crystals [30]. It is interesting that the crystals precipitated in the presence of L- or D-Asp can exhibit chiral morphology [31,32] as a consequence of the chiral structures of the enantiomers.

The existing literature has provided abundant knowledge on the effects of L-Asp on carbonate precipitation. Unfortunately, attempts have rarely been made for the possible application of L-Asp in sand reinforcement, and the investigation on shear properties is still insufficient [33,34]. Actually, the shear properties are the most basic and important mechanical properties for geotechnical materials [35]. Moreover, using L-Asp in sand reinforcement instead of cement can significantly promote the sustainable development goals of the construction industry concerning lower carbon emissions and energy consumption [36,37]. In this study, L-Asp was introduced into sand reinforcement through a newly developed technique of biomimetic carbonate precipitation (BCP) [38]. A series of direct shear tests were performed on BCP-treated sand with different numbers of spray cycles, and the impact of spray number on shear strength, residual strength, as well as critical displacement was investigated. Then, a simplified power model was established to describe the shear stress–displacement behavior of the specimens, and it was calibrated with the data obtained from tests. The work aims to provide a simple but effective model for the shear behavior of BCP-treated sand.

## 2. Methodology

### 2.1. Specimen Preparation

**Figure 1:**
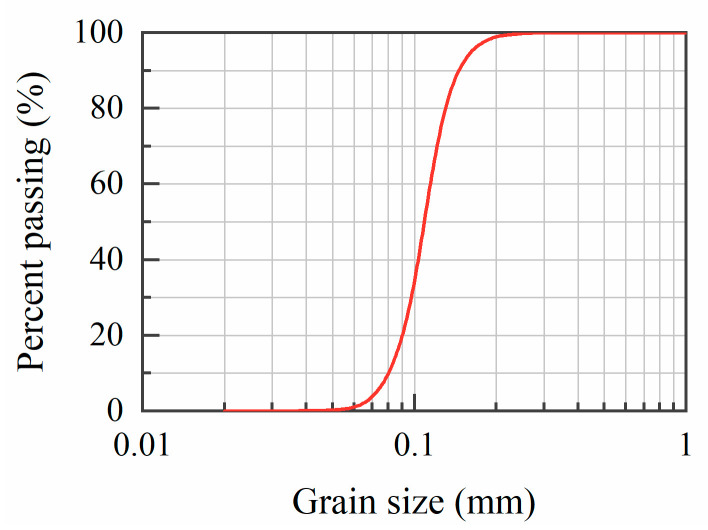
Gradation curve for Toyoura sand [33].

Specimens were prepared with Toyoura sand cemented by cementation solutions under room temperature. Toyoura sand was used as it is purer in composition (mainly SiO_2_), and the particle size is generally uniform. Detailed properties of Toyoura sand were given in Table 1, and the gradation was illustrated in Figure 1. Three cementation solutions were adopted, including C0 (CaCl_2_ solution with the concentration of 0.5 mol/L), C1 (mixture of CaCl_2_ and L-Asp, both of which are 0.5 mol/L), and C2 (Na_2_CO_3_ solution with a concentration of 0.5 mol/L). C0 and C1 were adjusted to be alkalescent (pH = 8.0) by a NaOH solution (1 mol/L), while C2 remained at its natural pH value. All the solutions were prepared with deionized water.

**Figure 2:**
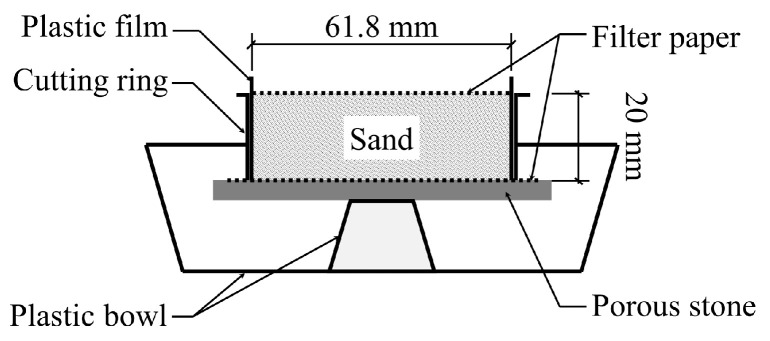
Mold for specimen preparation [33].

The molds for specimen preparation are exhibited in Figure 2. The bulk of the mold was a cutting ring with an inner diameter of 61.8 mm and a height of 20 mm. A thin plastic film was placed close to the inner surface of the cutting ring with both sides evenly coated with Vaseline for the convenience of demolding. Specimens were prepared according to the following procedures:

(1) By air pluviation, 85 g of completely dried sand was filled into the cutting ring.
(2) A piece of filter paper was placed on the top to prevent the deformation of specimen and make the fluid permeate evenly through the sand during the spray of cementation solutions. There was also a piece of filter paper at the bottom of the specimen in case the sand adhered to the porous stone.
(3) Deionized water was then sprayed on the filter paper covered on the specimen to get the sand saturated. After that, C1 (5 mL) was first applied from the top surface, and then C2 (also 5 mL) was applied, making one spray cycle.
(4) After a certain number of spray cycles, the cemented specimen was put into an oven of 105 °C for 24 h until the weight got constant.

In this study, four groups of specimens with different numbers of spray cycles were prepared, marked by labels from S1 to S4, respectively, in Table 2. A control group was also prepared with C0 and C2 through the same procedures and was labeled S0 in Table 2.

The final state of the specimen may be related to the number of spray cycles. With the increase in spray number, the structure of the specimen will undergo the following changes in sequence:

(1) CaCO_3_ precipitates and forms a coating on sand particles, which increases the friction between sand particles in the specimen.
(2) As more CaCO_3_ precipitates, the calcium coating gets thicker, and that on two adjacent sand particles finally gets in contact with each other to form a calcium bonding. That means all sand particles in the specimen have been bonded together by CaCO_3_ to form a continuous skeleton.
(3) The subsequent precipitation of CaCO_3_ starts to fill the pores in the skeleton to make the specimen denser.

It should be noted that CaCO_3_ can hardly fill all pores in the skeleton to make the material as compact as a solid material like concrete. Actually, all specimens obtained in this study are continuous porous material.

### 2.2. Direct Shear Test

A direct shear test is a basic test method for the mechanical properties of rocks and soils including sands. In this study, direct shear tests were performed on the specimens prepared in Section 2.1 with a TKA-DSS-4A four direct shear apparatus. For contrast, the direct shear tests were also conducted on pure sand. The shear rate was set to be 0.5 mm/min to ensure a quasi-static loading, and normal stress was 50 kPa.

For each specimen group, more than one specimen was prepared, and therefore multiple sets of shear stress–displacement relations could be obtained once loaded. Instead of selecting a specific set of data for analysis, we plotted all the data points together and used the midline of the upper and lower envelope to represent the shear stress–displacement relation of this group.

## 3. Results and Discussion

### 3.1. Effects of L-Asp Modifier

**Figure 3:**
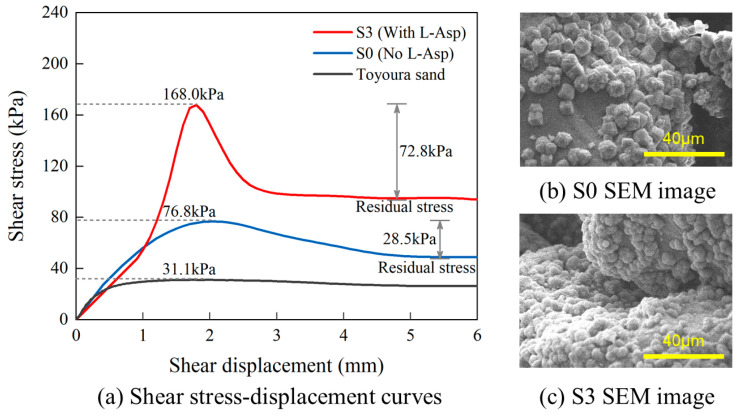
Shear stress–displacement relations and SEM images of S0 and S3.

The shear stress–displacement relations of groups S0 and S3 as well as pure sand have been plotted in Figure 3, and it can be found that both the strength of S0 and S3 are higher than that of the pure sand [38]. The shear strength of S0 is 76.8 kPa (average dispersion 5.9%), while that of S3 is 168.0 kPa (average dispersion 11.77%). Obviously, the specimen prepared with L-Asp has achieved a shear strength significantly higher than that without L-Asp, though the two groups have precipitated the same amount of CaCO_3_ in theory. The difference in strength can be attributed to the effects of L-Asp on the phase and morphology of carbonate precipitate. Calcium carbonate may have formed crystals and aggregated more orderly under the existence of L-Asp, thus leading to higher strength in the specimen. This inference has been confirmed by the scanning electron microscope (SEM) images presented in Figure 3, from which it can be seen that spherical vaterite had formed in the presence of L-Asp. The precipitated vaterite crystals are much denser than the rhombohedral calcite crystals formed without L-Asp, endowing the sand particles with stronger bonding between each other.

### 3.2. Effects of Spray Numbers

**Figure 4:**
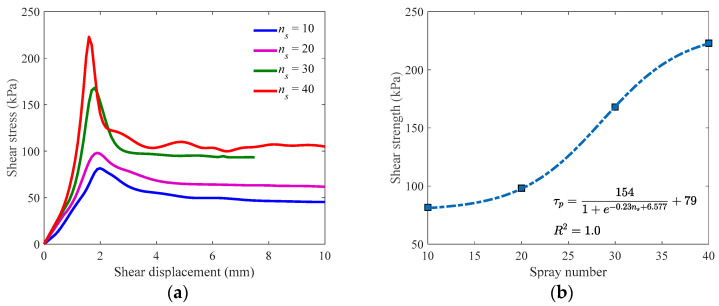

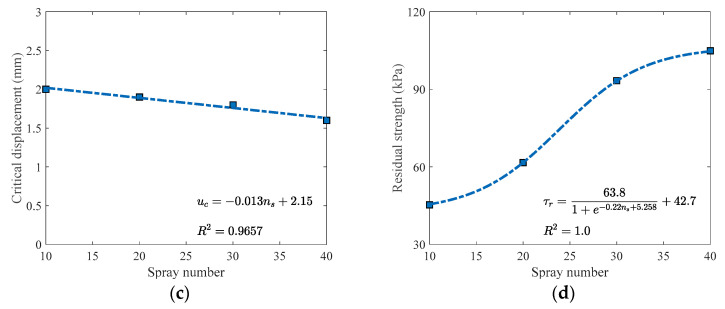
Correlation between different variables. (**a**) Shear stress-shear displacement; (**b**) Shear strength-spray number; (**c**) Critical displacement-spray number; (**d**) Residual strength-spray number.

The shear stress–displacement relations of specimens with different spray cycles are presented in Figure 4a [38], in which the number of spray cycles is denoted by $n_{s}$. It can be found that with the increase of spray number, the shear strength gets higher. However, such a variation is not linear. As shown in Figure 4b, the increase of shear strength at a lower or higher spray number is much slower than at the intermediate part. To better describe the relation between shear strength and spray number, a model in a sigmoid formula [39,40] is proposed herein as Equation (1), where $\tau_{p}$ is the shear strength, and p, w, k, and δ are all constants. The model was calculated with the test data, and Equation (2) could be obtained with an *R*^2^ value of 1.0. That means the proposed model fit the test results well.

(1) $$\tau_{p}=\frac{p}{1+e^{-wn_{s}+k}}+\delta$$

(2) $$\tau_{p}=\frac{154}{1+e^{-0.23n_{s}+6.577}}+79$$

Actually, the model has revealed an important fact that shear strength has an upper limit. As the spray number gets higher, shear strength also gets higher, but the increasing rate gradually slows down, and strength finally reaches the limit. According to Equation (1), such a limit is a constant p+δ. The variation of the model can be qualitatively explained as follows. When the spray number is small, the amount of L-Asp in the specimen is not enough for mediating carbonate precipitation. As the spray number increases, more precipitate could be mediated, and shear strength would also increase accordingly. But when the spray number is large enough, more L-Asp has been introduced into the specimen than the CaCO_3_ really needs, and the strength would never increase any more. The possible limit of the strength suggested by Equation (1) only happens when all the CaCO_3_ has been completely mediated. However, this can hardly be achieved in reality, since no chemical process could be expected to proceed completely. In this sense, a spray number of 30 seems to be suitable for engineering applications.

Define the shear displacement at which the shear stress reaches its peak as critical displacement (denoted by $u_{c}$), and it can be found from Figure 4a that critical displacement has shown an obvious decreasing trend as the number of spray cycles increases. That means the spray of cementation solutions would increase the brittleness of the specimen and in the meanwhile, endow the specimen with a higher modulus. Plot the critical displacement under different spray numbers in Figure 4c, and a linear correlation could be observed, which can be addressed as Equation (3) with an *R*^2^ value of 0.9657.

(3) $$u_{c}=-0.013n_{s}+2.15$$

Similar to shear strength, it is reasonable to assume the residual strength, which means the stable shear stress after the peak, also satisfies a sigmoid function as in Equation (1). Denote residual strength by $\tau_{r}$, and its variation with the spray number in a sigmoid formula can be calculated as Equation (4) with an *R*^2^ value of 1.0, as shown in Figure 4d.

(4) $$\tau_{r}=\frac{63.8}{1+e^{-0.22n_{s}+5.258}}\;+\;42.7$$

## 4. Simplified Model

### 4.1. Mechanics and Model Mathematics

**Figure 5:**
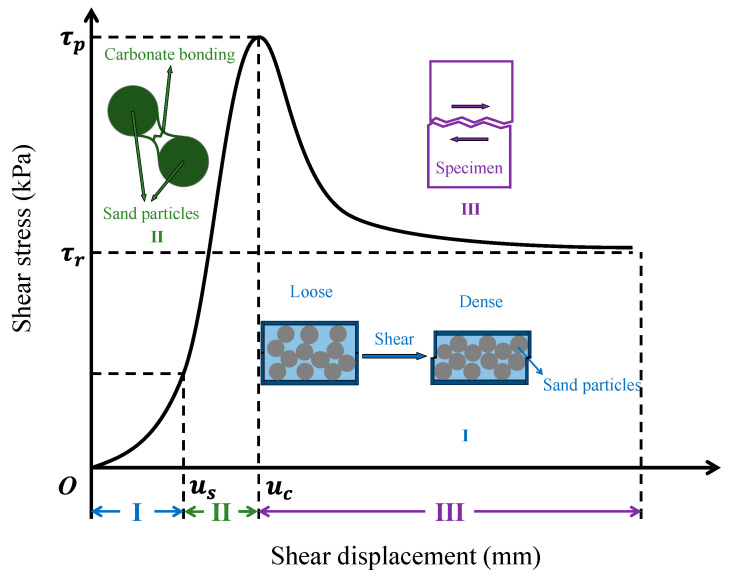
Division of different loading stages. I: Stage I; II: Stage II; III: Stage III.

The loading process can be divided into three stages, as illustrated in Figure 5. Specimen behavior in each stage is discussed as follows:

(1) Stage I ($0\leq u\leq u_{s}$)

Stage I is the integral shearing [41] of the specimen. In this stage, the sand skeleton bonded by CaCO_3_ is partially damaged under the coupling effect of normal stress and shear stress. As shear stress increases, sand particles rearrange and get more compacted. As a result, the modulus, or exactly, the tangent modulus, gets higher with the increase of shear displacement. Evidence could be found from the slope of the shear stress–displacement curve, which gradually increases with shear displacement. Such a phenomenon is also widely observed in the initial stage of direct shear loading on other materials formed by cemented sand, such as sandstone [42,43]. The strength of the specimen in this stage comes from two sources, one is the shear failure of the calcium bonding, and the other is the friction between the detached sand particles.

(2) Stage II ($u_{s}<u\leq u_{c}$)

Stage II starts following stage I and ends when the specimen fractures. In this stage, a shear band forms in the specimen, and the calcium bonding in the shear band is sheared to failure. As reflected by the stress–displacement curve, shear stress in this stage continuously increases, and the correlation between shear stress and displacement is roughly linear. With the formation of a shear band, shear stress starts to concentrate in the shear band, and the stress field transforms from uniform to non-uniform [41]. Friction also plays a role in this stage, but the strength comes more from the widespread damage of the calcium bonding in the shear band.

(3) Stage III ($u>u_{c}$)

The specimen enters Stage III once it fractures. In this stage, almost all calcium bonding in the shear band has been damaged, and the specimen has entered the critical state [44] where shear sliding starts to happen. Shear stress falls rapidly and finally stabilizes to a specific value. Stable stress, which has been defined as residual strength in the previous section, mainly comes from the friction between the upper and lower parts divided by the crack formed during the fracture of the specimen.

Actually, the three stages mentioned above are commonly found in the direct shear behavior of sand cemented by carbonate precipitation, such as microbially induced carbonate precipitation (MICP) [45,46,47].

To cover the three stages discussed above, a simplified model was established as Equation (5), in which both the monotonically increasing interval and the monotonically decreasing interval are described with power models. When displacement increases to be large enough, the model reduces to a constant, namely residual strength. Symbols a and b in Equation (5) are constants, and Δ is a tuning parameter calculated by Equation (6).

(5) $$\tau =\left\{\begin{array}{l} \begin{array}{cc} au^{b} & 0\leq u\leq u_{c} \end{array}\\ \begin{array}{cc} \max\left\{a{\left(2u_{c}-u+\Delta \right)}^{b},\tau_{r}\right\} & u_{c}<u<2u_{c}+\Delta \end{array}\\ \begin{array}{cc} \tau_{r} & u\geq 2u_{c}+\Delta \end{array} \end{array}\right.$$

(6) $$\Delta =k\left(u-u_{c}\right)$$

**Figure 6:**
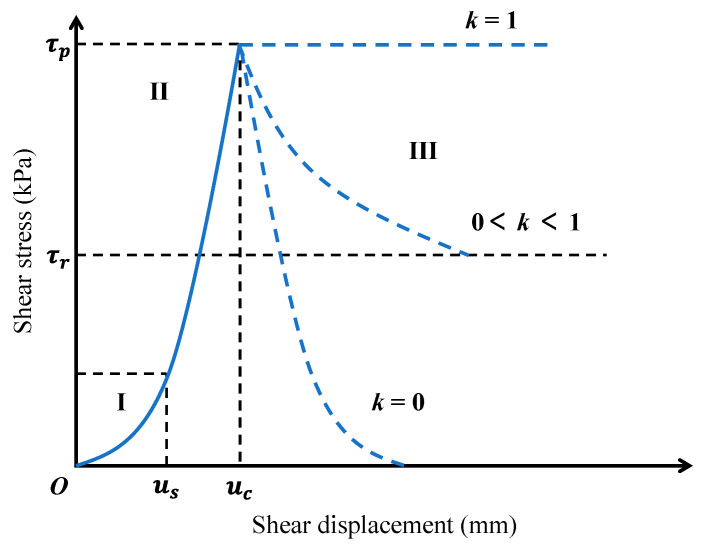
Simplified model under different *k* values. I: Stage I; II: Stage II; III: Stage III.

Constant k ($k\in \left[0,1\right]$) is adopted to adjust the trend of the power model in a monotonically decreasing interval. As illustrated in Figure 6, when k is set to be zero, the power model in a monotonically decreasing interval would be symmetric with the one in a monotonically increasing interval about the critical displacement. Meanwhile, when k is set to be one, the model reduces to a horizontal line passing through the peak point. If k is set to be a random number between 0 and 1, then the model is a curve falling in the region between the curves with k values of 0 and 1. Actually, the constant k has reflected the softening properties of the specimen in some degree. A k value of 1 represents a perfectly plastic state with no softening having occurred, while the value of 0 suggests significant post-peak softening. Therefore, the constant k is defined as the softening coefficient herein.

The curve shape of the power model in a monotonically increasing interval is controlled by constants a and b. Larger a and b will lead to a more rapid increase in shear strength, or more appropriately, the specimen having higher stiffness. Actually, the shear stiffness of the specimen can be characterized by the tangent modulus ($E_{t}$). As shown in Equation (7), for a given shear displacement u, the tangent modulus is largely decided by the product of a and b. In this sense, the value of a×b can reflect shear stiffness to a large extent.

(7) $$E_{t}\propto \frac{d\tau }{du}=abu^{b-1}$$

### 4.2. Calibration

**Figure 7:**
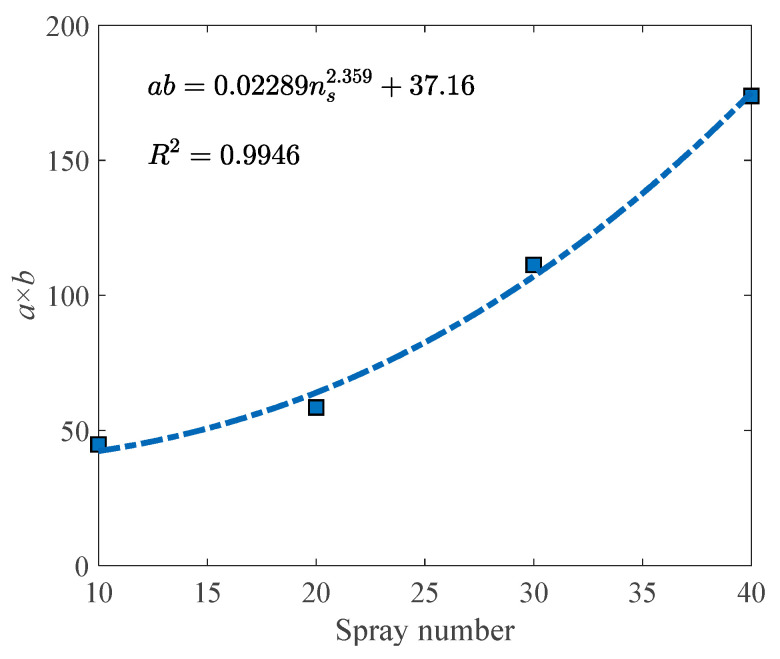
Correlation between spray number and the product of *a* and *b*.

Fit the measured data in a monotonically increasing interval with the proposed model, and constants a and b can be obtained. As discussed in Section 4.1, the shear stiffness of the specimen can be represented with the product of a and b. Therefore, the value of a×b was calculated, and the correlation illustrated in Figure 7 could be found. It can be observed from Figure 7 that as the spray number increases, the product of a and b also increases, and the growth rate gets faster and faster. That indicates that more spray cycles will lead to a significant increase in shear stiffness, which has been confirmed by the shear stress–displacement relations exhibited in Figure 4a.

(8) $$ab=0.02289{n_{s}}^{2.359}+37.16$$

**Figure 8:**
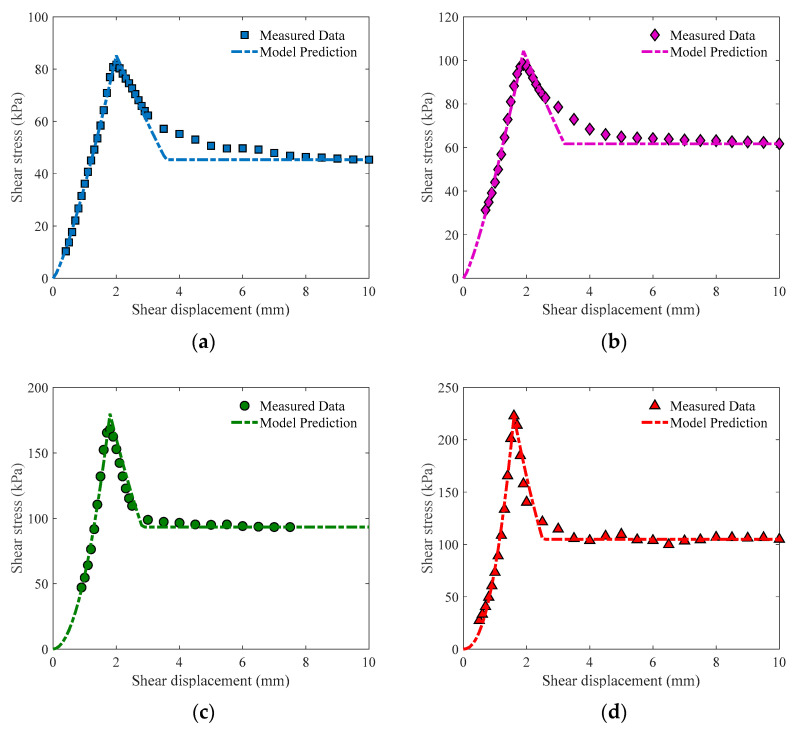
Model prediction under different spray numbers. (**a**) *n_s_* = 10; (**b**) *n_s_* = 20; (**c**) *n_s_* = 30; (**d**) *n_s_* = 40.

Set the softening coefficient k as 0.5, and models under different spray numbers can be plotted as in Figure 8. It can be found that the simplified model can give accurate predictions in most cases, especially at higher spray numbers. For post-peak shear stress, the estimate of the model is generally conservative. The value of 0.5 for coefficient k is enough for the model to perform well, indicating that the spray number has no obvious influence on the softening behavior of the specimen.

### 4.3. Application

For a given spray number ($n_{s}$), the model can be calculated according to the following steps.

(1) Calculate $\tau_{p}$, $u_{c}$, and $\tau_{r}$ with Equations (2), (3), and (4), respectively.
(2) With the obtained value of $u_{c}$ and $\tau_{p}$, there is Equation (8). Then, constants a and b can be solved from Equation (8) together with Equation (7).
  (9) $$a{u_{c}}^{b}=\tau_{p}$$
(3) Set k as 0.5, and the model is worked out.

## 5. Conclusions

In this study, a series of direct shear tests were performed on BCP-treated mortar, and the impact of spray number on shear strength, residual strength, as well as critical displacement was revealed. A simplified power model was then established for shear stress–displacement relations and calibrated with those obtained in tests. The conclusions are summarized as follows:

(1) BCP can significantly improve the shear strength of a sand specimen. As the number of spray cycles increases, the shear strength also increases, but the increasing rate at a moderate spray number is much higher than that at a lower or higher one. A similar variation was also observed for residual strength. The effects of spray number on shear strength and residual strength can both be described with sigmoid models, and a spray number of 30 is suggested for engineering applications.
(2) A BCP spray would increase both the brittleness and modulus of the specimen. As the spray number increases, critical displacement decreases, and the variation is roughly linear. Meanwhile, spray number seems to have no obvious influence on the softening behavior of the BCP-treated mortar.
(3) The simplified power model is well fitted to the three stages of direct shear loading and can give accurate predictions in most cases, especially at higher spray numbers. For post-peak stress, the estimate of the model is generally conservative. The softening coefficient *k* in the model is suggested to be 0.5, which is enough for the model to perform well.

## Figures and Tables

**Table 1:** Properties of Toyoura sand [38].

Apparent Density (g/cm^3^)	Packing Density (g/cm^3^)	Maximum Dry Density (g/cm^3^)	Minimum Dry Density (g/cm^3^)	Friction Angle (°)	D_50_ (mm)
2.654	1.430	1.603	1.386	31.39	0.13

**Table 2:** Different groups of specimens.

Labels	Compositions of Cementation Solutions (mol/L)			Spray Numbers
	CaCl_2_	L-Asp	Na_2_CO_3_	
S0	0.5	0.0	0.5	30
S1	0.5	0.5	0.5	10
S2	0.5	0.5	0.5	20
S3	0.5	0.5	0.5	30
S4	0.5	0.5	0.5	40

Note: Comparison between S0 and S3 can prove the effects of L-Asp in sand reinforcement, while with S1, S2, S3, and S4, the effects of spray number can be figured out.

## Data Availability

Data available on request from the authors.
